# Thyroid Function and Low Free Triiodothyronine in Chinese Patients With Autoimmune Encephalitis

**DOI:** 10.3389/fimmu.2022.821746

**Published:** 2022-02-10

**Authors:** Shan Qiao, Shan-chao Zhang, Ran-ran Zhang, Lei Wang, Zhi-hao Wang, Jing Jiang, Ai-hua Wang, Xue-wu Liu

**Affiliations:** ^1^ Department of Neurology, The First Affiliated Hospital of Shandong First Medical University and Shandong Provincial Qianfoshan Hospital, Jinan, China; ^2^ Department of Medical Genetics, School of Basic Medical Sciences, Cheeloo College of Medicine, Shandong University, Jinan, China; ^3^ Medical Research and Laboratory Diagnostic Center, Jinan Central Hospital, Cheeloo College of Medicine, Shandong University, Jinan, China; ^4^ School of Medicine, Cheeloo College of Medicine, Shandong University, Jinan, China; ^5^ Department of Neurology, Qilu Hospital, Cheeloo College of Medicine, Shandong University, Jinan, China; ^6^ Institute of Epilepsy, Shandong University, Jinan, China

**Keywords:** autoimmune encephalitis, thyroid function, free triiodothyronine, low-T3 syndrome, prognosis

## Abstract

**Background and Objectives:**

Low free triiodothyronine (FT3) is usually associated with worse functional outcome in critical illness; however, the information on thyroid dysfunction and autoimmune encephalitis (AE) is limited. This study aims to evaluate the clinical prognostic value of thyroid function and low-T3 syndrome in patients with multiple subtypes of AE.

**Methods:**

In this retrospective study, we identified the hospital records of 319 candidate patients with AE admitted between January 2016 and December 2020. We then extracted the clinical features and outcomes. Modified Rankin scale (mRS) scores were used to evaluate the patients’ neurological function. The serum levels of FT3, free thyroxine (FT4), and thyroid-stimulating hormone (TSH) were measured upon admission. Normal thyroid stimulating hormone level with FT3 below the lower limit of the reference interval (2.63 nmol/L) was defined as low-T3 syndrome.

**Results:**

A total of 237 AE cases remained after screening. Among these, 57.81% (137/237) were men and the average age at onset was 41 y (interquartile range, 12–61 y). We found that 83.54% (198/237) of the patients had a good prognosis, and 16.46% (39/237) had a poor prognosis. Abnormal thyroid function was observed in 30.80% of these patients, with a relatively greater prevalence in the group with a poor prognosis (*p* < 0.001). The serum FT3 levels in the poor-prognosis group were significantly lower than those in the good-prognosis group (*p* < 0.001). Low-T3 syndrome occurred in 15.19% of AE cases and was more frequent in patients with poor prognosis (p < 0.001).

**Conclusions:**

Abnormal thyroid function in AE is frequent, and serum FT3 levels in patients with poor prognosis are significantly lower than in those with good prognosis. Low-T3 syndrome could be a potential candidate for predicting the prognosis of AE following future research.

## Introduction

Autoimmune encephalitis (AE) is a severe inflammatory brain disorder mediated by autoimmune mechanisms and accounts for 10% to 20% of encephalitis cases (1). The main clinical features of AE are acute or subacute epilepsy, cognitive dysfunction, and mental abnormalities. Considering cases of rapidly progressive cognitive dysfunction and encephalitis of unknown etiology, AE occupies an extremely important place as a treatable disease (2, 3). As of 2007, encephalitis owing to anti-N-methyl-D-aspartate receptor (anti-NMDAR) was the most frequent AE subtype, followed by the subtypes due to anti-leucine-rich glioma-inactivated 1 (anti-LGI1) and anti-gamma aminobutyric acid B-receptor (anti-GABABR) antibody. Other antibody-defined subtypes include those due to anti-contactin-associated protein-like 2 (anti-CASPR2), anti-α-amino-3-hydroxy-5-methyl-4-isoxazole propionate receptor (anti-AMPAR), anti-myelin oligodendrocyte glycoprotein (anti-MOG), anti-glutamic acid decarboxylase 65(anti-GAD 65), and others. Most patients respond well to immunotherapy; however, some remain with intractable seizures and varying degrees of cognitive impairment (4). Early diagnosis and treatment have a positive effect on the prognosis but are difficult to ensure since the clinical manifestations and prognosis vary widely across subtypes and some patients are antibody-negative. Therefore, exploring new biological markers useful for AE diagnosis and prognosis would ensure better treatment effectiveness.

Thyroid hormones, which mainly comprise triiodothyronine (T3) and thyroxine (T4), play a crucial role in the development and maturation of the mammalian central nervous system (5). Free T3 (FT3) is the active component of thyroid hormone and directly reflects the functional status of the thyroid gland. Previous studies have suggested that low FT3 levels are closely related to the prognosis of neurological diseases such as cognitive dysfunction, brain tumors, neuromyelitis optica spectrum disorder, and ischemic stroke. Low-T3 syndrome, which is characterized by decreased serum T3, decreased or normal serum tetraiodothyronine (T4), and normal thyroid-stimulating hormone (TSH) levels, is usually associated with worse functional outcomes, poor prognosis, and greater mortality in critical illness (6, 7). The presence of anti-thyroid antibodies reportedly correlates with worse outcomes in patients with anti-NMDAR encephalitis (8). Another study of 94 patients concluded that low total thyroxine had predictive value for the adverse outcomes in severe encephalitis (9). A 2018 study by Ma et al. that included 43 patients with anti-NMDAR encephalitis revealed that low-T3 syndrome could predict longer hospitalization and greater clinical severity (10). Accumulating evidence suggested that low-T3 syndrome could be a potential candidate for the prediction of the prognosis of AE. However, to our knowledge, few large-scale clinical studies have focused on the predictive value of low-T3 syndrome in the progression of AE.

Therefore, we aimed to investigate whether thyroid dysfunction, especially low-T3 syndrome, is associated with the clinical manifestations and prognosis of AE in a sample of patients in which multiple subtypes were represented.

## Materials and Methods

### Study Design and Patient Selection

This retrospective study was conducted at Qilu Hospital, Cheeloo College of Medicine, Shandong University, China, from January 2016 to December 2020, and initially included 319 patients diagnosed with AE according to published diagnostic criteria (3). The inclusion criteria were as follows: (1) acute or subacute onset of one or more clinical features, including seizures, memory deficit, mental and behavioral disorder, and speech disturbance related to the limbic system, (2) serum and/or cerebrospinal fluid (CSF) positivity for neuron-surface antibodies, and (3) reasonable exclusion of other disorders. The exclusion criteria were as follows: (1) history of intrinsic thyroid disorder (hypothyroidism, hyperthyroidism, thyroiditis, or central hypothyroidism, which are mostly caused by hypothalamic or pituitary diseases/conditions), (2) comorbidities that could directly cause low-T3 syndrome, including serious heart failure, acute myocardial infarction, severe hepatic disease, and renal failure, (3) medication with anti-thyroid drugs and/or thyroid hormone replacement or receiving therapy with drugs known to affect the secretion and metabolism of thyroid hormones, and (4) incomplete clinical data. Eighty-two patients were excluded.

The modified Rankin scale (mRS) scores were used to assess the effects of the treatment and clinical outcomes. We followed up the patients every 2–3 months throughout the first year after discharge and every 4–6 months thereafter. Patients were followed up for at least 1 year. The follow-up data were carefully retrieved from the hospital records or by interviewing (directly or by telephone and WeChat) the patients and their families. At 6 months after discharge, patients with an mRS score ≤2 were defined as the good-prognosis group, and patients with an mRS score >2 were defined as the poor-prognosis group.

### Standard Protocol Approvals, Registrations, and Patient Consents

This study was approved by the Ethics Committee of Qilu Hospital of Shandong University (approval number: KYLL-202008-044) and was conducted in accordance with the Declaration of Helsinki. Written, informed consent was obtained from all study participants or their legal guardians.

### Data Collection and Diagnostic Criteria for Low-T3 Syndrome

We recorded the baseline demographics, clinical data, treatment information, patient prognosis, and the results of the auxiliary examinations, including CSF analyses, serum analyses, EEG, and brain MRI. Autoantibodies to NMDAR, LGI1, GABA_B_R, CASPR2, AMPA1, and AMPA2 were assessed in all 319 patients *via* indirect immunofluorescence (Euroimmun, Germany). Approximately one-quarter of the patients were tested for paraneoplastic antibodies, including anti-Hu, anti-Yo, anti-Ri, and anti-amphiphysin. The initial dilution titers of serum and CSF were 1:10 and 1:1, respectively. Blood samples for thyroid function testing were drawn from all the patients within 24 h of the first hospital admission. Thyroid hormone levels were measured in our hospital using a chemiluminescent analyzer (Cobas E601, Shanghai, China). According to the manufacturer’s instructions, the reference intervals were 2.63–5.70 pmol/L for serum FT3, 9.01–19.05 pmol/L for serum free T4 (FT4), and 0.350–4.94 μIU/mL for thyroid stimulating hormone (TSH). Patients with normal TSH levels but with FT3 below the lower limit of the reference interval (FT3 < 2.63 nmol/L) were defined as having low-T3 syndrome.

### Statistical Analysis

SPSS IBM 26.0 and GraphPad Prism 8.0 software were used to perform statistical analyses. Normally distributed continuous variables are presented as means and standard deviations. Non-normal data are presented as medians and interquartile ranges (IQR). Categorical variables are described as percentages. Data were analyzed using the *χ* ^2^ test or Fisher’s exact test, and the Mann–Whitney U test was used for continuous variables. Spearman’s correlation coefficient was used to identify any correlations between low-T3 syndrome and clinical characteristics. Two-sided values of *p* < 0.05 were considered significant.

## Results

### Baseline Demographics and Clinical Features of Patients With AE

**Figure 1:**
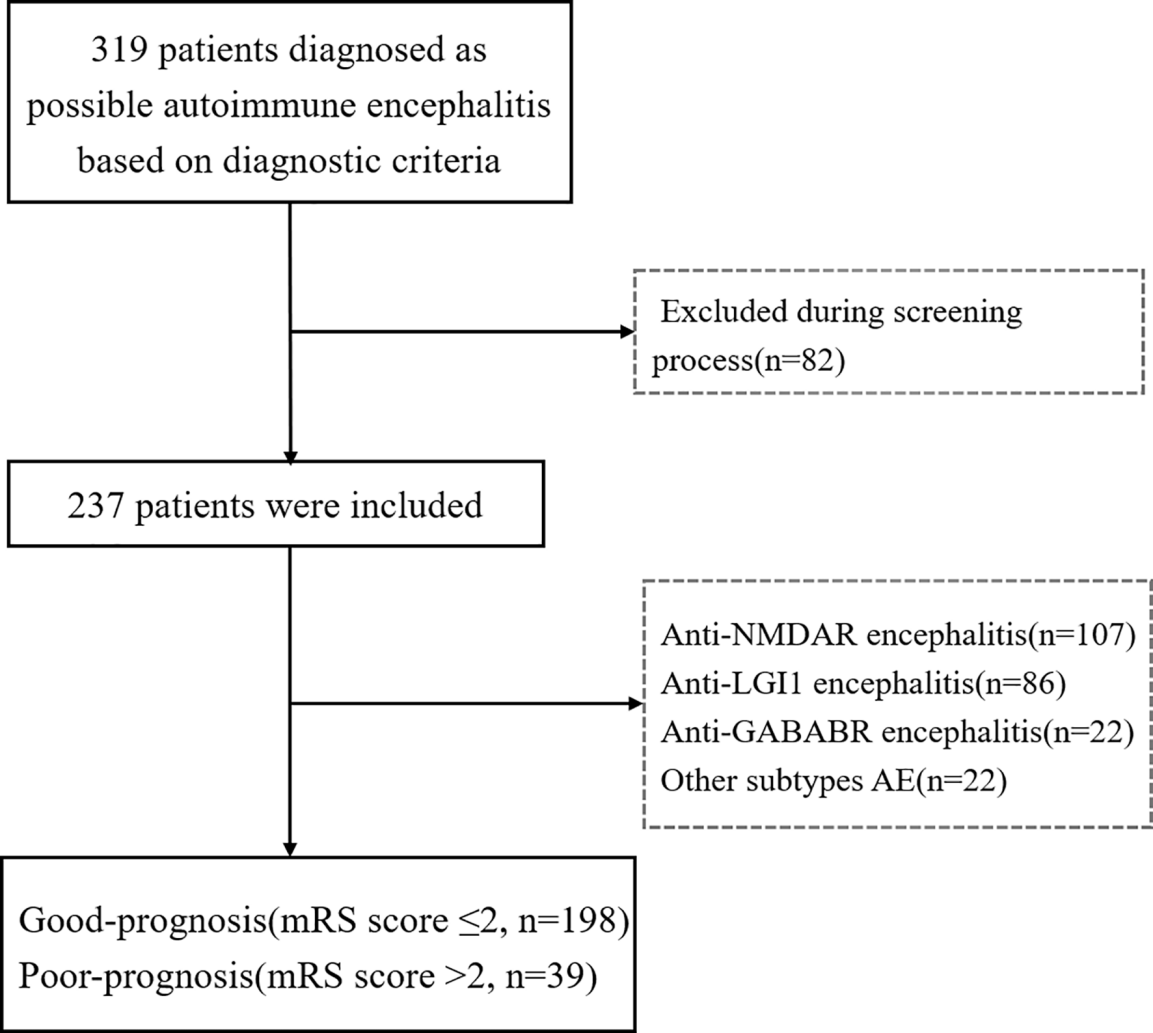
The flowchart of patient recruitment. mRS, modified Rankin scale.

The included cases comprised 107 patients with anti-NMDAR encephalitis, 86 with anti-LGI1 encephalitis, 22 with anti-GABABR encephalitis, and 22 with miscellaneous subtypes of AE; these being anti-CASPR2 encephalitis (9 cases), anti-AMPAR encephalitis (4), anti-MOG encephalitis (6), anti-GAD 65 encephalitis (2), and anti-amphiphysin encephalitis (1). Patient recruitment is described in the flowchart in **Figure 1**. Of the 237 patients included in this study, 57.81% (137/237) were men, and the average age at onset was 41 years (IQR, 12–61). According to the mRS score at 6 months after discharge, 83.54% (198/237) of patients had a good prognosis, and 16.46% (39/237) had a poor prognosis (**Table 1**). No fatalities were recorded. Nine patients died within 2 y of discharge from severe pneumonia, tumor, or other complications. These cases comprised seven with anti-GABABR encephalitis and two with anti-NMDAR encephalitis.

**Table 1:** Demographics and clinical features of patients with autoimmune encephalitis.

Variable	Total (n=237)	Good-prognosis (n=198)	Poor-prognosis (n=39)	*p*
Age at onset, y, median, IQR	41 (12–61)	44 (16–62)	22 (17–47)	0.109
Sex, Male/Female	137/100	123/75	14/25	0.002*
Subtype of AE antibody (n, %)				<0.001*
Anti-NMDAR	107 (45.15)	79 (39.90)	28 (71.79)	
Anti-LGI1	86 (36.29)	84 (42.42)	2 (5.13)	
Anti-GABAR	22 (9.30)	17 (8.59)	5 (12.82)	
Others	22 (9.30)	18 (9.09)	4 (10.26)	
Clinical Symptoms (n,%)				
Seizures	170 (71.73)	133 (67.17)	37 (94.87)	0.001*
Memory deficit	162 (68.35)	132 (66.67)	30 (76.92)	0.208
Mental behavioral disorders	135 (56.96)	100 (50.51)	35 (89.74)	<0.001*
Disturbance of consciousness	97 (40.93)	62 (31.31)	35 (89.74)	<0.001*
Movement disorder	70 (29.54)	42 (21.21)	28 (71.79)	<0.001*
Central hypoventilation	16 (6.75)	4 (2.02)	12 (30.77)	<0.001*
Complicated with tumors	22 (9.28)	12 (6.06)	10 (25.64)	<0.001*
mRS scores at admission	3.78 ± 0.80	3.56 ± 0.68	4.87 ± 0.34	<0.001^†^
Ancillary tests results (n,%)				
Increased level of protein in CSF	72 (30.38)	30 (15.15)	12 (30.77)	0.954
Hyponatremia	74 (31.22)	66 (33.33)	8 (20.51)	0.114
Abnormal thyroid function	73 (30.80)	50 (25.25)	23 (58.97)	<0.001^†^
FT3	3.68 ± 0.060	3.84 ± 0.06	2.85 ± 0.14	<0.001^†^
FT4	13.46 ± 0.20	13.57 ± 0.21	12.93 ± 0.55	0.229
TSH	1.84 ± 0.15	1.87 ± 0.17	1.66 ± 0.22	0.330
Low T3 syndrome	36 (15.19)	14 (7.07)	22 (56.41)	<0.001*
Abnormal brain MRI signal	129 (54.43)	106 (53.54)	23 (58.97)	0.533
Abnormal EEG findings	160 (67.51)	132 (66.67)	22 (56.41)	0.105
Treatment (n, %)				
Steroids	227 (95.78)	189 (95.45)	38 (97.44)	0.574
IVIG	189 (79.75)	155 (78.28)	34 (87.18)	0.206
Steroids + IVIG	178 (75.11)	143 (72.22)	35 (89.74)	0.021*
Second-line treatment	35 (14.77)	22 (11.11)	13 (33.33)	<0.001*

*χ2, *p value in Fisher’s exact test; ^†^ p value in Student’s t test, and values are presented as means ± standard deviation.

IQR, interquartile range; AE, autoimmune encephalitis; anti-NMDAR, anti-N-methyl-D-aspartate receptor; anti-LGI1, anti-leucine-rich glioma-inactivated 1; anti-GABABR, anti-gamma aminobutyric acid B-receptor; mRS, modified Rankin scale; CSF, cerebrospinal fluid; FT3, free triiodothyronine; FT4, free thyroxine; TSH, thyroid stimulating hormone; MRI, magnetic resonance imaging; EEG, electroencephalogram; IVIG, intravenous immunoglobulin,

As described in **Table 1**, the most frequent clinical manifestations of AE in our sample were seizures, memory deficits, mental and behavioral disorder, and disturbance of consciousness. Patients with a good prognosis had lower mRS scores at admission than did patients with a poor prognosis. Anti-NMDAR and anti-GABAR encephalitis were more common in the poor-prognosis group, accounting for 71.79% and 12.82%, respectively (*p*< 0.001). In addition, among patients with poor prognosis, the following conditions were more frequent: seizures, mental and behavioral disorder, disturbance of consciousness, movement disorder, central hypoventilation, and complicated with tumors; these differences were statistically significant (*p* < 0.05).

Auxiliary test findings are described in **Table 1**. In our sample, 30.38% of patients had elevated CSF protein levels and 31.22% had hyponatremia; however, the difference between the good- and poor-prognosis groups was not significant. Moreover, 54.43% showed abnormal brain MRI results, with focal lesions mainly in the hippocampus, temporal lobes, and frontal lobes. Abnormal electroencephalogram (EEG) findings of unilateral or bilateral nonspecific slow waves were observed in 67.51% of the patients.

Of note, 30.80% of the sample showed at least some abnormal thyroid function, with a greater prevalence in the poor-prognosis group. Further, the serum FT3 levels of patients with a poor prognosis were significantly lower than those of patients with a good prognosis. No significant difference was observed between the two groups in the serum FT4 or TSH levels. Low-T3 syndrome occurred in 15.19% of patients with AE and was more prevalent in patients with a poor prognosis.

Of the total sample, 95.78% were treated with steroids, 79.75% were treated with intravenous immunoglobulin (IVIG), and 75.11% were treated with steroids + IVIG; 14.77% of the patients had received second-line treatments, namely rituximab, cyclophosphamide, mycophenolate mofetil, or azathioprine. A relatively greater percentage of patients with a poor prognosis were treated with combined steroids and IVIG, and a relatively greater percentage underwent second-line treatment.

### Clinical Characteristics Stratified by T3 Status

The demographic, clinical, and biological characteristics of patients with and without low-T3 syndrome are described in **Table 2**. In our sample, 15.19% of the patients (13 males, 23 female) had low-T3 syndrome, with FT4 and TSH levels within or below the normal ranges at diagnosis. Another 201 cases (124 male, 77 female) did not have low-T3 syndrome. Notably, patients with low-T3 syndrome had higher mRS scores at admission than did patients without low-T3 syndrome. The AE subtype composition in the 36 patients with low-T3 syndrome was as follows: 26, anti-NMDAR; 6, anti-LGI1; 2, anti-GABAR; and 2, antibodies defining other subtypes (1, anti-amphiphysin; 1, anti-AMPAR).

**Table 2:** Clinical characteristics of patients with autoimmune encephalitis with or without low T3 syndrome.

Variable	low T3 syndrome (n=36)	Without low T3 syndrome (n=201)	*p*
Age at onset, y, median, IQR	28.50 (18–57.25)	44 (16–62)	0.508
Sex, Male/Female	13/23	124/77	0.004*
mRS scores at admission	4.61 ± 0.55	3.63 ± 0.75	<0.001**^#^**
Subtype of AE antibody (n, %)			0.005*
Anti-NMDAR	26 (72.22)	81 (40.30)	
Anti-LGI1	6 (16.67)	80 (39.80)	
Anti-GABAR	2 (5.56)	20 (9.95)	
Others	2 (5.56)	20 (9.95)	
Clinical Symptoms (n, %)			
Seizures	34 (94.44)	136 (67.66)	0.001*
Memory deficit	22 (61.11)	140 (69.65)	0.310
Mental behavioral disorders	31 (86.11)	104 (51.74)	<0.001*
Disturbance of consciousness	28 (77.78)	69 (34.33)	<0.001*
Movement disorder	22 (61.11)	48 (23.88)	<0.001*
Central hypoventilation	12 (33.33)	4 (2.00)	<0.001*
Complicated with tumors	8 (22.22)	14 (6.97)	0.004*
Ancillary tests results			
Increased level of protein in CSF (n, %)	14 (38.89)	58 (28.86)	0.228
Hyponatremia (n, %)	13 (36.11)	61 (30.35)	0.492
FT3, pmol/L	2.23 ± 0.062	3.94 ± 0.052	<0.001^†^
FT4, pmol/L	12.52 ± 0.44	13.63 ± 0.23	0.045^†^
TSH, μIU/mL	1.70 ± 0.31	1.86 ± 0.16	0.257
Abnormal brain MRI signal (n, %)	23 (63.89)	106 (52.74)	0.216
Abnormal EEG findings (n, %)	20 (55.56)	140 (69.65)	0.096
Treatment (n, %)			
Steroids	24 (66.67)	193 (96.02)	0.665
IVIG	30 (83.33)	159 (79.10)	0.561
Steroids + IVIG	30 (83.33)	148 (73.63)	0.215
Second-line treatment	10 (27.78)	25 (12.44)	0.017*
Prognosis (n,%)			<0.001*
Good-prognosis	14 (38.89)	184 (91.54)	
Poor-prognosis	22 (61.11)	17 (8.46)	
mRS scores at 6 months after discharge	2.72 ± 0.94	1.42 ± 0.71	<0.001^#^

*χ2, *p value in Fisher’s exact test.

^†^p value in Student’s t test.

Values are presented as means ± standard deviation.

^#^Z value in the Mann-Whitney U-test.

IQR, interquartile range; AE, autoimmune encephalitis; anti-NMDAR, anti-N-methyl-D-aspartate receptor; anti-LGI1, anti-leucine-rich glioma-inactivated 1; anti-GABABR, anti-gamma aminobutyric acid B-receptor; mRS, modified Rankin scale; CSF, cerebrospinal fluid; FT3, free triiodothyronine; FT4, free thyroxine; TSH, thyroid stimulating hormone; MRI, magnetic resonance imaging; EEG, electroencephalogram; IVIG, intravenous immunoglobulin.

The percentages of seizures, mental and behavioral disorder, disturbance of consciousness, movement disorder, central hypoventilation, and tumors were significantly greater in patients with low-T3 syndrome than in those without ow-T3 syndrome. We observed no significant differences in the percentages of other symptoms or clinical characteristics, including memory deficit, increased levels of protein in the CSF, hyponatremia, abnormal brain MRI signal, or abnormal EEG findings.

In patients with low-T3 syndrome, the average values of serum FT3, FT4, and TSH were 2.23 ± 0.062 pmol/L, 12.52 ± 0.44 pmol/L, and 1.70 ± 0.31 μIU/mL, respectively. In cases without low-T3 syndrome, the corresponding levels were 3.94 ± 0.052 pmol/L, 13.63 ± 0.23 pmol/L, and 1.86 ± 0.16 μIU/mL. The differences between the two groups in FT3 and FT4 levels were significant.

A relatively greater proportion of patients with low-T3 syndrome were treated with combined immunotherapy using steroids + IVIG. In addition, 10 patients with low-T3 syndrome were treated with second-line immunotherapy, a significantly greater percentage than among patients without. The percentage of patients with poor prognosis with low-T3 syndrome was significantly greater than that of patients without low-T3 syndrome (*p* < 0.001).

### Association Between Low-T3 Syndrome and Clinical Characteristics

As shown in **Table 3**, sex, mRS scores at admission, seizures, mental and behavioral disorder, disturbance of consciousness, movement disorder, central hypoventilation, complicated with tumors, and mRS scores at 6 months following discharge were all significantly and inversely associated with FT3 levels, while subtype of AE antibody was positively associated with the FT3 levels. However, no significant correlations were detected between FT3 levels and the following clinical features: age at onset, memory deficit, increased levels of protein in the CSF, hyponatremia, abnormal brain MRI signal, and abnormal EEG findings.

**Table 3:** Correlation analysis of possible factors for low T3 syndrome in patients with autoimmune encephalitis (n=237).

Predictive variables	*R*	*p*
Sex	-0.186	0.004**
Age at onset	0.035	0.508
mRS scores at admission	-0.449	<0.001**
Subtype of AE antibody	0.194	0.001**
Seizures	-0.213	0.001**
Memory deficit	0.066	0.311
Mental behavioral disorders	-0.249	<0.001**
Disturbance of consciousness	-0.317	<0.001**
Movement disorder	-0.293	<0.001**
Central hypoventilation	-0.448	<0.001**
Complicated with tumors	-0.18	0.004**
Increased level of protein in CSF	-0.078	0.229
Hyponatremia	-0.045	0.493
Abnormal brain MRI signal	-0.080	0.217
Abnormal EEG findings	0.108	0.097
mRS scores at 6 months after discharge	-0.501	<0.001**

^*^p < 0.05, ^**^p < 0.01.

AE, autoimmune encephalitis; mRS, modified Rankin scale; CSF, cerebrospinal fluid; MRI, magnetic resonance imaging; EEG, electroencephalogram.

## Discussion

The present study included 237 patients with multiple subtypes of AE, and we found that 30.80% exhibited abnormal thyroid function in the acute phase (onset). In addition, we focused on the relationship between low-T3 syndrome and AE. We found that in addition to anti-NMDA encephalitis, low-T3 syndrome was also present in anti-LGI1 encephalitis, anti-GABAR encephalitis, and other rare subtypes of encephalitis, suggesting that low-T3 syndrome may be prevalent in multiple subtypes of AE.

To the best of our knowledge, few studies have investigated the relationship between low-T3 syndrome and AE. Our findings suggest that patients with poor prognosis have a higher prevalence of this syndrome than the good-prognosis group. In particular, the serum FT3 levels of patients with poor prognosis were significantly lower than those of the good-prognosis group. We further explored the association between low-T3 syndrome and clinical characteristics in patients with AE.

Reports of abnormal thyroid function in AE are common and have gradually attracted the attention of researchers (11). Previous studies have focused on the association between anti-NMDA encephalitis and thyroid function and anti-thyroid antibodies but had a limited sample size. Large studies and studies on the relationship between other subtypes of encephalitis and thyroid function are rare. Most of these investigations are single case reports (12–14). Nevertheless, considering the important role of thyroid hormones in the development of the nervous system and in maintaining normal physiological function, this phenomenon is of sufficient importance to warrant the attention of researchers. In September 2021, it was reported that anti-thyroid antibodies (ATAbs) were more frequent in patients with severe disease than in the non-severe group, and that ATAbs were not only prevalent in patients with anti-NMDAR encephalitis but correlated with poor prognosis (15). Another study of 51 cases (Chen et al.) retrospectively analyzed the relationship between anti-NMDAR encephalitis and thyroid function, reporting that ATAbs and abnormalities in FT3, FT4, and TSH levels were frequent in pediatric anti-NMDAR encephalitis (16). In our previous study (17) of 117 patients with anti-LGI1 encephalitis, we found that abnormal thyroid function was frequent in this group: 31.6% (37/117) showed abnormal thyroid function, and 22% of these had elevated levels of serum thyroid peroxidase antibodies. In another multicenter study (18) that included 185 patients with multiple subtypes of AE, 63 (34.05%) had abnormal thyroid function. However, to the best of our knowledge, studies on multiple subtypes of AE and thyroid function are rare.

The pathogenesis of low-T3 syndrome remains unclear and is thought to be related to impaired peripheral thyroid hormone metabolism, hypothalamic-pituitary-thyroid axis dysfunction, decreased levels of thyroid binding globulin, the involvement of multiple cytokines, and an altered internal milieu (5, 19–21). Low-T3 syndrome is frequent in neurological diseases and plays an important role in the assessment of the condition and its prognosis (6, 22). The finding of the presence of low-T3 syndrome in multiple subtypes of AE promises to provide new ideas to further elucidate the pathogenesis of AE.

T3 mainly participates in the catabolic pathways of the body, and the decline in T3 levels observed in severe disease serves to protect the body by conserving energy (10, 23). Low-T3 syndrome can thus be viewed as a useful adaptation in the acute phase of critical illness (5, 19, 23). Therefore, it is controversial whether thyroid hormone supplementation should be administered in the acute phase of severe disease accompanied by low-T3 syndrome (20, 24). In addition, we here found a significant negative correlation between mRS scores (greater representing more severe disease) at admission and FT3 levels, suggesting that patients with AE with low FT3 might have relatively severe disease in the acute phase. However, since this study was retrospective and the thyroid hormone levels were all measured in the acute phase and thereby did not reveal dynamic changes over the disease course, the association of the FT3 levels with the period of AE remission remains unclear. Prospective studies are therefore warranted to further elucidate the causal relationship between low-T3 syndrome and AE.

This study also focused on the characteristics of patients with AE and low-T3 syndrome. We found that patients with AE with low-T3 syndrome were more likely to have seizures, mental disorder, disturbed consciousness, movement disorder, central hypoventilation, and tumors, and these factors were significantly and negatively associated with low FT3. We also found that among patients with AE with poor prognosis, these symptoms were more frequent. This is consistent with the results of previous studies. In a 2018 study (8) that included 43 patients with anti-NMDAR encephalitis, the authors found that low-T3 syndrome occurred frequently in anti-NMDAR encephalitis (25.6%) and concluded that low FT3 levels are also associated with a decline in consciousness and with the mRS score on admission. In another study of anti-NMDAR encephalitis (16), Chen et al. found that ATAbs were more frequent in patients with severe disease than the control group (51.4% vs 25.6%), and the prevalence of ATAbs was associated with a higher incidence of central hypoventilation, and disturbance of consciousness. Of note, patients with ATAbs had lower FT3 levels. Accumulating evidence suggests that low-T3 syndrome is also related to poor outcomes in patients with various neurological disorders such as ischemic stroke (22), brain tumor (25), and neuromyelitis optica spectrum disorder (aka NMOSD) (26). In our study, the results demonstrated that anti-NMDAR and anti-LGI1 encephalitis were more common in patients with AE with low-T3 syndrome, accounting for 72.22% and 16.67%, respectively (< 0.005), which are consistent with previous studies. To date, to our knowledge, no research has been conducted on the relationship between anti LGI1 encephalitis and thyroid hormone. In our study, 6 (6.98%) of 86 patients with anti LGI1 encephalitis were found to have low-T3 syndrome in the acute stage. There may be differences in the incidence of low-T3 syndrome among the different subtypes of AE. The clinical significance of these differences warrants in-depth studies with a larger sample size, which may provide clues for the pathogenesis of the disease.

Low T3 syndrome is not considered a lesion of the thyroid itself, but a decrease in the circulating thyroid hormone levels caused by serious diseases (21). The thyroid hormone level can gradually return to normal follwoing the recovery of the patients’ basic diseases upon treatment. Accumulating evidence suggests that lower serum T3 concentrations may be associated with greater severity, more complicated clinical course, greater mortality rates and elevated risk for poor functional outcomes at discharge and long term, including patients with acute cerebrovascular events, patients with respiratory failure, and after surgery for brain tumor (27). Our results suggested that patients with AE with low FT3 could have relatively severe disease at onset, and the mRS scores of patients with AE at 6 months following immunotherapy were inversely associated with FT3 levels, which were similar to those in previous studies, which could suggest that low T3 syndrome is related to the disease severity in the acute stage of AE to a certain extent, and has a certain early cautionary effect on its prognosis. However, the current evidence does not yet indicate the exact long-term predictive value of low-T3 levels in the acute phase of AE. This is also a limitation of the current study. Future studies with a larger sample size are needed to clarify this.

According to previous studies, there may be a correlation between the thyroid hormone level and IgG subclasses (5, 24). In addition, the dynamic changes of thyroid hormone IgG levels are related to the condition and prognosis of a variety of immune diseases to a certain extent (8). Owing to the different indicators for different diseases affected by the patient’s condition, economic perspective, patient’s willingness, and physician’s choice preference, only 41 patients recorded complete thyroglobulin, anti-thyroglobulin antibodies, and anti-thyroid peroxidase autoantibody data in our cohort of 237 patients. Due to the limited sample size and large individual differences, we were not able to clarify the clinical significance of the thyroid hormone level and IgG subclasses in AE. This is a further limitation of the current research. The study reflected to some extent the characteristics of patients with AE in the east of China; the geographical limitations of the regional design limit the variability of the demographic characteristics, thereby limiting the ability to generalize the findings. In addition, the current study is retrospective and has limitations in terms of completeness of patient data and monitoring of dynamic changes in disease. Therefore, large sample, multi-center, prospective, and longitudinal studies are needed; we will continue to pay attention to the correlation between thyroid hormone level and the long-term prognosis of AE.

## Conclusions

The current study focused on thyroid function and low-T3 syndrome in patients with multiple subtypes of AE, and we are aware of few comparable studies. The results suggested that low-T3 syndrome may be prevalent in multiple subtypes of AE, and that low T3 might be related to a more severe disease state in the acute phase. This suggests the possibility that thyroid hormones are of great importance in the pathogenesis of AE, providing clues for future studies aiming to elucidate the pathogenesis of AE and improve diagnosis. Larger samples and prospective studies are warranted to clarify the relationship between low-T3 syndrome and AE prognosis.

## Data Availability Statement

The raw data supporting the conclusions of this article will be made available by the authors, without undue reservation.

## Ethics Statement

The studies involving human participants were reviewed and approved by Ethics Committee of Qilu Hospital of Shandong University. Written informed consent to participate in this study was provided by the participants’ legal guardian/next of kin.

## Author Contributions

X-wL and A-hW conceived this study and participated in its design. SQ organized the data and drafted the manuscript. S-cZ searched the literature and organized the data. R-rZ, LW, Z-hW, and JJ assisted in the data collection. All the authors have read and approved the final version of the manuscript.

## Funding

The National Natural Science Foundation (No. 81873786) supported this work.

## Conflict of Interest

The authors declare that the research was conducted in the absence of any commercial or financial relationships that could be construed as a potential conflict of interest.

## Publisher’s Note

All claims expressed in this article are solely those of the authors and do not necessarily represent those of their affiliated organizations, or those of the publisher, the editors and the reviewers. Any product that may be evaluated in this article, or claim that may be made by its manufacturer, is not guaranteed or endorsed by the publisher.
